# Effect of variation in ovine *WFIKKN2* on growth traits appears to be gender-dependent

**DOI:** 10.1038/srep12347

**Published:** 2015-07-22

**Authors:** Jiqing Wang, Huitong Zhou, Qian Fang, Xiu Liu, Yuzhu Luo, Jon G. H. Hickford

**Affiliations:** 1 Gansu Key Laboratory of Herbivorous Animal Biotechnology, Gansu Agricultural University, Lanzhou 730070, China; 2 Faculty of Animal Science and Technology, Gansu Agricultural University, Lanzhou 730070, China; 3 Gene-Marker Laboratory, Faculty of Agriculture and Life Sciences, Lincoln University, Lincoln 7647, New Zealand

## Abstract

WFIKKN2 may play a role in the regulation of muscle growth and development, but to date there have been no reports on the effect of variation in *WFIKKN2* on growth and carcass traits in livestock. In this study, the effect of variation in ovine *WFIKKN2* was investigated in 800 New Zealand Romney lambs (395 male and 405 female), with five previously described variants (*A* to *E*) being identified. Variation in ovine *WFIKKN2* was not found to affect various growth traits in the female lambs, but the presence of variant *B* was associated (*P* < 0.05) with decreased birth weight, tailing weight, weaning weight and pre-weaning growth rate; and increased post-weaning growth rate in male lambs. In male lambs, the presence of variant *B* was associated (*P* < 0.05) with an increased shoulder yield and proportion shoulder yield. No associations with growth or carcass traits were detected for the presence (or absence) of the other variants. These results suggest that variation in ovine *WFIKKN2* may have a differential effect on growth in male and female lambs, and hence that the gene may be expressed in, or act in, a gender-specific fashion.

Myostatin (also known as growth and differentiation factor 8, GDF8) is a regulator of myogenesis and acts primarily as a negative regulator of muscle growth in mammals. Deletions or mutations in the myostatin gene (*MSTN*) cause an increase in skeletal muscle mass as a result of a combination of hypertrophy (an increase in the size of muscle fibres) and hyperplasia (an increase in the number of muscle fibres). This effect has been recorded in sheep^1^, cattle^2^,^3^,^4^, dogs^5^, mice^6^ and humans^7^.

Investigations into the action of myostatin have led to the discovery of other proteins that affect its activity. These include WFIKKN1 (also called growth and differentiation factor associated serum protein-2, GASP2) and WFIKKN2 (also called GASP1). Both are large extracellular multi-domain proteins consisting of a WAP (whey acidic protein)-domain, a Follistatin-domain, an immunoglobulin-domain, two Kunitz-type protease inhibitor-domains and a netrin-domain^8^.

It has been revealed experimentally that the WFIKKN1 and WFIKKN2 proteins can inhibit the biological activity of myostatin by binding to the extracellular domain (ECD) of the myostatin receptor protein^9^,^10^,^11^,^12^. For example, using surface plasmon resonance assays in a solution-competition format, Szlama *et al*.^11^ revealed that 50 nM WFIKKN1 and WFIKKN2 caused an 80% and 90% decrease respectively in the rate of association of myostatin with its receptor ECD. In this assay the ECD of the myostatin receptor is immobilized on a chip surface and myostatin in solution pre-incubated with increasing concentrations of WFIKKN is added. The chip can detect receptor binding. The observation that WFIKKN2 was found to have a higher affinity than WFIKKN1 for the myostatin receptor, suggests that WFIKKN2 is a more effective receptor agonist^10^.

In addition to myostatin, WFIKKN2 also inhibits the biological activity of growth and differentiation factor 11 (GDF11)^11^. This factor also affects muscle growth and development, and GDF11 knock-out mice exhibit skeletal defects resulting from abnormal anterior-posterior patterning^13^. Studies have revealed that *WFIKKN2* has a high level of expression in the skeletal muscle of mice^9^,^14^,^15^ and in developing human foetal tissues^16^.

Up to now, research into the effect of WFIKKN2 on muscle growth and development has primarily been in mice. For example, Monestier *et al*.^17^ revealed that over-expression of *WFIKKN2* in a transgenic mouse model caused increased muscle growth that was consistent with inhibition of myostatin activity. What is more, the viral delivery of a *WFIKKN2* expression cassette into the muscle of adult mice has been shown to induce increases in muscle mass^18^. Lee & Lee^15^ further confirmed the effect of *WFIKKN2* on muscle weight, when describing how mice lacking *WFIKKN2* had a shift in muscle fibre type from fast glycolytic type IIb fibres, to fast oxidative type IIa fibres, and that they had impaired muscle regeneration ability.

Little is known about WFIKKN2 or its targets in livestock. However, in 2012 a patent was granted to the Huazhong Agricultural University in China for an invention in which the pig WFIKKN2 gene is described as a molecular marker for carcass characteristics^19^. Little else is known about the underlying science in this patent.

Based on the above reports, it could be concluded that *WFIKKN2* might be a candidate gene controlling variation in growth and carcass muscle traits in meat-producing animals. However, very little is known about the gene or its product, and whether they are variable in livestock species. What is more, most of the SNPs found to date in the gene of humans, pigs, cattle and chickens are derived from the Ensembl and GenBank databases and have been identified by comparison of different DNA sequences. The presence of these SNPs has not been described in published scientific literature.

Recently Wang *et al*.^20^ revealed five variants and twelve SNPs in intron 1 of ovine *WFIKKN2* using PCR-SSCP. The objective of this study was to analyse if associations exist between the variation identified by Wang *et al*.^20^ and growth and carcass muscle traits in New Zealand (NZ) Romney lambs.

## Results

### Variant and genotype frequencies of *WFIKKN2* in NZ Romney lambs

All of the five variants of *WFIKKN2* described previously by Wang *et al*.^20^ were found in the sheep studies. The frequencies of the five variants in the lambs are shown in Table 1. In both genders, *A* and *B* were the most common variants, followed by *C*, *D* and *E*. Genotypes *AA*, *AB*, *BB*, *AC* and *BC* were the more common genotypes with a total frequency of 93.93% in the male lambs, and 93.83% in the female lambs investigated. The remaining genotypes (*CC*, *AD*, *BD*, *CD*, *AE*, *BE*, *CE* and *DE*) only occurred with a total frequency of less than 7% in lambs of both gender (data not shown). A chi-square test revealed that there was no difference in variant frequencies between the male and female lambs (*P* = 0.984; Table 1).

For the common variants *A*, *B* and *C*, there was a significant difference between the expected and actual genotype frequencies in the male lambs (*P* = 0.002; Table 2). Genotypes *AA* and *BB* were less common than expected and *AB* was more common, while no significant differences for genotypes *AC*, *BC* and *CC* were observed between the actual and expected genotype frequencies. This effect was not observed for the females lambs (*P* = 0.994; Table 2).

### Associations between variation in ovine *WFIKKN2* and variation in growth traits in male and female NZ Romney lambs

Of the five variants identified, association analyses between the genetic variation and variation in growth and carcass muscle traits were restricted to variants *A*, *B* and *C*; as *D* and *E* were present at a frequency less than 2% and this could potentially confound the analyses.

First, the relationship between variation in ovine *WFIKKN2* and variation in growth traits was tested in male lambs, and it was found that the presence (or absence) of *A* and *C* was not found to have an effect on those traits (Table 3). Moreover, the *P* values for these analyses were greater than 0.2 in the single-variant models, and therefore these were not factored into the multi-variant models.

The models revealed that the presence of *B* in a genotype was associated with decreased birth weight (present: 5.3 ± 0.07 kg; absent: 5.6 ± 0.12 kg; *P* = 0.036), decreased tailing weight (present: 18.3 ± 0.25 kg; absent: 19.5 ± 0.46 kg; *P* = 0.020), decreased weaning weight (present: 32.2 ± 0.35 kg; absent: 33.9 ± 0.64 kg; *P* = 0.012) and decreased pre-weaning growth rate (present: 298 ± 3 g/d; absent: 313 ± 6 g/d; *P* = 0.023), whereas it was associated with increased post-weaning growth rate (present: 239 ± 9 g/d; absent: 191 ± 16 g/d; *P* = 0.005) (Table 3).

No significant association was found between the presence of *B* and draft weight and growth rate from birth to draft.

Association of the presence (or absence) of ovine *WFIKKN2* variants with growth traits was investigated in the female lambs, but no associations were found (Table 3).

### Associations between variation in ovine *WFIKKN2* and variation in carcass muscle traits in male NZ Romney lambs

In the single-variant presence/absence models, the presence of *B* was associated with increased shoulder yield (present: 17.0 ± 0.09%; absent: 16.8 ± 0.13%; *P* = 0.043) and proportion shoulder yield (present: 32.0 ± 0.07%; absent: 31.7 ± 0.13%; *P* = 0.035) (Table 4).

The presence of *B* also tended to be associated with decreased proportion leg yield (present: 40.5 ± 0.07%; absent: 40.8 ± 0.13%; *P*=0.057) and the presence of *C* tended to be associated with decreased proportion shoulder yield (present: 31.7 ± 0.14%; absent: 32.0 ± 0.08%; *P* = 0.078) (Table 4).

In the multi-variant presence/absence models, the effect of *B* on proportion shoulder yield was lost when *C* was introduced into models; but a trend was still evident (*P* = 0.095; Table 4). The trend for *C* to be associated with decreased proportion shoulder yield was lost when *B* was introduced into models.

No associations with any carcass muscle traits were detected for the presence (or absence) of *A* (Table 4).

## Discussion

This is the first study to report associations between variation in ovine *WFIKKN2* and variation in growth and carcass muscle traits. In total, five variants previously defined by Wang *et al*.^20^ were detected in this study, with no new variants being found. Of the five variants, *A* and *B* were the most common with a total frequency of more than 86% in lambs of both genders. This may be a consequence of sire selection, as a majority of the sires (eight were *AB*, one was *BB*, two were *BC*, one was *BD* and one was *AC*) were heterozygous for the *AB* genotype.

Although the SNPs identified in this study are not located in the coding regions of *WFIKKN2*, they may be linked to other variation in critically important regions of the gene that regulate expression. The variation is located in intron 1 which could possess important regulatory elements, such as enhancers, silencers or other elements, and these could modify expression level of their host gene in many different ways^21^,^22^,^23^. Alternatively this intron may affect mRNA levels and protein yield by affecting virtually any step of mRNA maturation; including transcription initiation, elongation and termination, polyadenylation, nuclear export and mRNA stability^24^,^25^.

Given that a significant difference between the actual and expected genotype frequencies for *AA*, *AB* and *BB* only existed in male lambs, and that variation in ovine *WFIKKN2* was only associated with variation in growth traits in the male NZ Romney lambs, but not in the female lambs; it could therefore be inferred that WFIKKN2's activity may be gender-specific, or gender-preferential. In this context, in transgenic mice with muscle-specific over-expression of *MSTN*, Reisz-Porszasz *et al*.^26^ found a gender-specific phenomenon, where muscle-mass difference from wild-type was only detected in male mice, but not in female mice. They suggested it may be as a consequence of a gender-specific mechanism that overrides the effects of *MSTN* on muscle mass in female transgenic mice. Whether this mechanism is a consequence of WFIKKN2 activity can only be speculated upon.

It is also notable that Han *et al*.^27^ reported an abnormal gender-ratio for ovine *MSTN* variation in NZ Romney sheep, suggesting the gene may be affected by some gender-specific mechanism. Given WFIKKN2 interacts with myostatin via the latter’s receptor, the gender-specific mechanisms observed for *MSTN* may actually reflect variation in *WFIKKN2* expression, and a subsequently the downstream myostatin receptor binding of WFIKKN2. This too is only speculation, but the contention is supported by the observation that gender-specific differences in muscle weight have been described by Lee & Lee^15^ in both 10-week-old and 8-month-old *WFIKKN2* transgenic mice. At 10 weeks of age, there was a significant decrease in the weight of the gastrocnemius muscle in *WFIKKN2*^*−/−*^ male mice compared with age-matched wild-type mice. This was not observed for the female mice. At 8 months of age, a decrease in muscle weight for the triceps was only observed in the *WFIKKN2*^*−/−*^ male mice.

The WFIKKN2 gene is expressed in the ovaries and testes in adult humans^12^, suggesting the gene may be involved in the regulation of sex hormones. Testosterone, as the most important androgen, is mainly synthesized by the Leydig cells of testes in males (95%) and a small amount is produced by the ovaries in females. Testosterone induces skeletal muscle hypertrophy due to protein accumulation and myonuclear accretion in humans^28^,^29^. In sheep there are strong correlations between testosterone concentration and carcass lean content^30^,^31^, bone content^32^ and body weight and growth rate^33^. WFIKKN2 may therefore also potentially have different effects on growth in male and female lambs, via a testosterone-mediated mechanism.

It has been reported that the individual weights of pectoralis major muscles were increased in a gain-of-function transgenic mouse model that overexpresses *WFIKKN2*^17^. This is consistent with the effect reported by Lee & Lee^15^, where wild-type mice had an increased muscle weight for their triceps than *WFIKKN2*^−/−^ mice. A higher proportion of muscle mass in the shoulder region is a secondary sexual characteristic of rams and is thus considered responsive to testosterone^34^,^35^. Similar effects have also been described in humans. Having broader shoulders, a hallmark effect of puberty in boys, has been positively correlated with testosterone level^36^.

In the context of the above argument, the association between variation in ovine *WFIKKN2* and shoulder yield and proportion shoulder yield reported here, may be related to variation in the expression of the gene in the testis, where it affects testosterone activity and therefore may be involved in the onset of puberty in male lambs after weaning. It is noteworthy that the variation described in ovine *WFIKKN2* differentially affects shoulder yield, but not leg and loin yield in the carcasses studied, suggesting a male-specific muscle maturation mechanism. Caution is needed in this interpretation though, as in the *WFIKKN2* transgenic mouse model, the weight of gastrocnemius and rectus femoris muscles also changed^17^.

In this study, variation in ovine *WFIKKN2* was found to have a tendency to affect proportion leg yield (*P* = 0.072). In humans, narrower hips are another masculine trait besides having broader shoulders and this is related to testosterone production^37^. In sheep, a smaller proportion of leg muscle in male lambs, than in female lambs, has been described^38^. Other literature also reports a tendency for a decrease in proportion of leg muscle in male lambs, although no significant differences from female lambs have been described^39^,^40^.

It must also be noted that in this study the effect of the presence of variant *B* on total yield is small. This is consistent with the findings of Lee & Lee^15^ and Monestier *et al*.^17^ in mice. One potential explanation for the small phenotypic effect in these sheep, is that all the sires from which the lambs were derived in this study were ranked in the top of 20% of NZ Romney rams in New Zealand based on SIL-DPO breeding index value (http://www.sil.co.nz), and thus they have arguably already been selected for high productivity. There may therefore only be a small variation in carcass muscle traits for the lambs studied relative to other NZ Romney sheep, or other breeds. This conclusion is supported by Hickford *et al*.^41^, who only detected a small effect of *MSTN* variation on skeletal muscle mass in NZ Romney lambs. These lambs had also been produced by rams in the top 20% for SIL-DPO.

In mice it is also suggested that WFIKKN2 may have some functional redundancy, with other proteins, such as follistatin (FST), also capable of regulating muscle development^15^. It has been reported that there is an increase in muscle mass in transgenic mice over-expressing *FST*, an effect that was larger than the effect observed in *MSTN*^−/−^ mice^42^. The possibility exists therefore that there may also be some functional redundancy between WFIKKN2 and FST or other regulatory proteins in sheep. Accordingly, it might be important to investigate the effect of WFIKKN2 on carcass muscle traits in combination with a study of FST or other regulatory proteins.

Given that the effect of *WFIKKN2* variation on carcass muscle traits appears to be small, it could be concluded that the utility of *WFIKKN2* in marker-assisted selection for improved carcass muscle traits would be of little value. However, further study is necessary to validate these results in more sheep and of other breeds, prior to drawing this conclusion.

A chi-square test showed that there was a significant difference between the expected and actual genotype frequencies for the common variants *A*, *B* and *C* in the male lambs (*P* = 0.002), but not in the female lambs (*P* = 0.994). The difference may be related to lamb mortality. For bovine *MSTN*, unequal genotype distributions have been observed and the difference between actual and expected numbers has been shown to be related to calves dying^43^,^44^. The reason for the gender-specific genotype distributions in this study may be because female lambs are at a lower risk of mortality pre-natally and peri-natally, compared to their male counterpart^45^.

Variation in ovine *WFIKKN2* was only associated with growth traits in the male lambs, but not in the female lambs. The presence of *B* was associated with decreased pre-weaning growth traits, but increased post-weaning growth rate. In the New Zealand sheep industry, weaning weight is considered a critically important criterion for deciding when to slaughter lambs and thus selecting the lambs with the absence of *B* could result in increased economic benefits. This approach might therefore have some value as a way for improving growth performance in male lambs, especially if used in conjunction with a terminal-sire breeding system.

It is notable that of all the growth traits investigated in this study, the presence of *B* had no effect on draft weight and growth rate from birth to draft. This is not surprising though, as draft dates were arbitrarily set and a threshold weight was set for slaughter. After 20 weeks all remaining male lambs were slaughtered regardless of weight, so in the context of the selection based on different criteria at different times, the failure to find an association is perhaps unsurprising.

Associations between variation in ovine *MSTN* and growth and carcass muscle traits in NZ Romney lambs attracted our attention due to the biological interaction of its encoded protein with WFIKKN2. Hickford *et al*.^41^ reported variation in *MSTN* was associated with leg yield, loin yield and total yield in lambs, but not shoulder yield and various growth traits. The effect of *WFIKKN2* appears to contrast these findings, with its variation only associated with shoulder yield and growth traits. Together these studies suggest that both genes can affect carcass meat yield, but they may work to either augment or suppress each other depending on which variant is expressed, in which gender and at what stage of maturity.

Up to now, the precise mechanism of the effect of WFIKKN2 on muscle development is unknown. The only well-documented finding is that WFIKKN2 could play a role in the regulation of muscle development by inhibiting the biological activity of myostatin and GDF11. However WFIKKN2 could also regulate muscle mass via another pathway or through other proteins. For example, papilin, a protein influencing cell rearrangement in muscle tissue^46^,^47^, has been shown to share a common origin and similar protein structure with WFIKKN2^48^ and it gives a new perspective to analyse WFIKKN2 activity in muscle development. Furthermore, cyclin D1 (Ccnd1), activin A receptor type IC (Acvr1c), myosin heavy chain (Myh3), myogenic differentiation 1 (Myod1) and peroxisome proliferator activated receptor γ (Pparγ) are reportedly involved in muscle development, and are down- or up-regulated in transgenic *WFIKKN2* mice^17^. This suggests WFIKKN2 could potentially interact with at least five other proteins in the process of muscle development. Finally, in view of it possessing a follistatin-domain, WFIKKN2 protein may have an overlapping function with follistatin and thus regulate muscle growth via the effect of follistatin^49^.

In conclusion, the present study suggests that ovine *WFIKKN2* may be expressed in a gender-specific fashion and that variation in ovine *WFIKKN2* appears to have an effect on growth traits, and a small effect on carcass traits, but only in male lambs.

## Materials and Methods

All research involving animals were carried out in accordence with the Animal Welfare Act 1999 (New Zealand Govertment) and the collection of sheep blood drops by nicking sheep ears is covered by Section 7.5 Animal Identification, of the Animal Welfare (Sheep and Beef Cattle) Code of Welfare 2010; a code of welfare issued under the Animal Welfare Act 1999 (New Zealand Government).

### Sheep investigated and data collection

A total of 395 male lambs and 405 female lambs produced by thirteen un-related NZ Romney rams were investigated. The male lambs only were slaughtered for meat production and were used to investigate associations between variation in *WFIKKN2* and variation in both growth and carcass muscle traits, while the female lambs that were kept as flock replacement were only used to analyse associations with growth traits.

All the lambs were ear-tagged with a unique identification number within 12 h of birth and the birth date, birth rank (i.e. whether they were a single, twin or triplet), birth weight and gender were recorded. All the lambs were tailed at 2–6 weeks of age and weighed at that time.

The lambs were weaned at approximately 12 weeks of age, weighed and separated according to gender. Pre-weaning growth rate for both the male and female lambs was calculated as the difference between weaning weight and birth weight, divided by their age in days (expressed in grams/day). At weaning, the male lambs weighing 37 kg or more, were drafted for slaughter. The remaining male lambs were next weighed at 16 weeks of age and those that had then reached 37 kg were slaughtered. Finally at 20 weeks of age all remaining male lambs were slaughtered by the Alliance Group Limited (http://www.alliance.co.nz/RP.jasc?Page=Home). Draft age and weight were recorded for each male lamb and their post-weaning growth rate was calculated as the difference between draft weight and weaning weight, divided by age in days (expressed in grams/day). An overall, growth rate from birth to draft for each male lamb was calculated as the difference between draft weight and birth weight, divided by age in days (expressed in grams/day).

Hot carcass weights (H-W) were measured directly on the processing chain. H-W is the weight in kilograms of the carcass minus the pelt, head and gut. Video image analysis (VIASCAN; Sastek), developed by Meat and Livestock Australia and described in Hopkins *et al*.^50^, was used to estimate the following carcass muscle traits: lean meat yield (expressed as a percentage of H-W) in the leg (leg yield), loin (loin yield) and shoulder (shoulder yield), total yield (the sum of the leg, loin and shoulder yields for any given carcass), the proportion leg yield, the proportion loin yield and the proportion shoulder yield. The proportion yield of leg, loin or shoulder is the yield of the specific area divided by the total yield, expressed as a percentage.

Of the 395 male and 405 female lambs used for identification of variation in intron 1 of ovine *WFIKKN2*, a small number of lambs were excluded from the association analyses as a result of having incomplete data. Accordingly sample numbers may vary slightly in the different analyses.

### Sheep blood samples and DNA extraction

At tailing, a blood sample from each lamb was collected onto an FTA card (Whatman BioScience, Middlesex, UK) by a skilled operator (JGHH). Genomic DNA from all the sheep studied was purified according to the method described by Zhou *et al*.^51^.

### PCR amplification and SSCP analysis

A 421-bp fragment of intron 1 of ovine *WFIKKN2* was amplified using PCR primers 5′-GAGACGGACCAGGTGAGTG-3′ and 5′-AGAGGCTGGATGAAGCATCG-3′. Amplifications were performed in a 20 μL reaction consisting of the DNA on one 1.2 mm punch of FTA card, 2.0 μL of 10 × PCR buffer, 0.25 μM of each primer, 150 μM dNTPs, 2.5 *m*M Mg^2+^, 0.5 U *Taq* DNA polymerase (Qiagen, Hilden, German), and ddH_2_O to make up volume. The thermal profile consisted of 2 min at 94 °C, followed by 35 cycles of 30 s at 94 °C, 30 s at 58 °C and 30 s at 72 °C, with a final extension of 5 min at 72 °C.

For SSCP analysis, a 0.7 μL aliquot of each amplicon was mixed with 7 μL of loading dye (98% formamide, 10 *m*M EDTA, 0.025% bromophenol blue, 0.025% xylene-cyanol) and after denaturation at 95 °C for 5 min, samples were rapidly cooled on wet ice and then loaded on 16 × 18 cm, 14% acrylamide: bisacrylamide (37.5:1) (Bio-Rad, Hercules, CA, USA) gels. Electrophoresis was performed using Protean II xi cells (Bio-Rad) for 19 h in 0.5 × TBE at 220 V at 25 °C. Gels were silver-stained according to the method of Byun *et al*.^52^.

### Statistical analyses

Expected values for each genotype of ovine *WFIKKN2* for both the male and female lambs separately were calculated based on actual variant frequencies using POPGENE version 3.2. The frequency of the genotypes (actual and expected) in both the male and female lambs was analyzed using a chi-square test.

The data from all thirteen Romney sire-lines were pooled. Linear Mixed-Models were used to estimate whether the presence or absence (coded as 1 or 0 respectively) of a particular variant in a genotype was associated with growth and carcass muscle traits. The associations between variation in ovine *WFIKKN2* and variation in growth traits were analysed separately in the male and female lambs. Association analyses were performed using SPSS version 16.0.

In the models, variant presence or absence was fitted as a fixed factor and sire was fitted as a random factor. For growth traits, birth rank was fitted as a fixed factor, while for the carcass muscle traits, either birth rank was fitted as a fixed factor, or birth weight was fitted as a covariate, and depending on which had a bigger effect on individual carcass muscle traits. The single-variant presence/absence models were performed for each *WFIKKN2* variant to ascertain which variant should be included in subsequent multi-variant models, such that we could assess whether the effect of the single variant was independent of the other variant in the genotype. The subsequent multi-variant models included any variant as a factor that had an association with a trait in the single-variant models with a *P* value of less than 0.200 (and which could therefore also potentially impact on trait).

Unless otherwise indicated, all *P* values were considered statistically significant when *P* < 0.05 and trends were noted when 0.05 < *P* < 0.10.

## Additional Information

**How to cite this article**: Wang, J. *et al*. Effect of variation in ovine *WFIKKN2* on growth traits appears to be gender-dependent. *Sci. Rep*. **5**, 12347; doi: 10.1038/srep12347 (2015).

## Figures and Tables

**Table 1:** Frequency of ovine *WFIKKN2* variants in NZ Romney lambs.

Gender	*n*	Frequency (%)					*P* value^1^
		*A*	*B*	*C*	*D*	*E*	
Male	395	38.73	47.59	11.52	1.39	0.77	0.984
Female	405	38.64	47.65	11.36	1.60	0.75	

^1^*P* value was derived form a chi-square test for frequency differences when comparing male and female lambs.

**Table 2:** Ovine *WFIKKN2* genotype frequencies in male and female lambs and their deviation from expected values^1^.

Genotype^2^	Male lambs		Female lambs	
	Actual (%)	Expected^2^ (%)	Actual (%)	Expected^2^ (%)
*AA*	9.11	15.00	15.56	14.93
*AB*	50.13	36.86	36.54	36.82
*BB*	15.70	22.65	23.21	22.71
*AC*	8.10	8.92	7.90	8.78
*BC*	10.89	10.96	10.62	10.83
*CC*	1.77	1.33	1.48	1.29
*P* value^3^	0.002		0.994	

^1^Genotypes containing the two rare variants (*D* and *E*) are not shown.

^2^Expected value for the specific genotype was calculated using POPGENE version 3.2.

^3^*P* value was derived from a chi-square test for genotype frequency difference (i.e. between the expected and actual values).

**Table 3:** Association of ovine *WFIKKN2* variants with growth traits (mean ± SE)^1^ in male and female NZ Romney lambs.

Growth trait	Variant	Male lambs					Female lambs				
		Variant absent	*n*	Variant present	*n*	*P* value^1^	Variant absent	*n*	Variant present	*n*	*P* value^1^
Birth weight (kg)	*A*	5.3 ± 0.10	119	5.4 ± 0.07	262	0.504	5.1 ± 0.09	145	5.0 ± 0.07	245	0.603
	*B*	5.6 ± 0.12	75	5.3 ± 0.07	306	**0.036**	5.0 ± 0.10	105	5.1 ± 0.07	285	0.788
	*C*	5.4 ± 0.07	300	5.4 ± 0.13	81	0.956	5.1 ± 0.07	310	5.0 ± 0.11	80	0.664
Tailing weight (kg)	*A*	18.4 ± 0.37	119	18.6 ± 0.27	262	0.684	17.6 ± 0.31	145	17.2 ± 0.25	245	0.301
	*B*	19.5 ± 0.46	75	18.3 ± 0.25	306	**0.020**	17.3 ± 0.35	105	17.4 ± 0.24	285	0.838
	*C*	18.7 ± 0.26	300	18.1 ± 0.49	81	0.337	17.3 ± 0.24	310	17.5 ± 0.39	80	0.716
Weaning weight (kg)	*A*	32.3 ± 0.52	119	32.6 ± 0.38	262	0.593	29.8 ± 0.43	145	29.6 ± 0.34	245	0.730
	*B*	33.9 ± 0.64	75	32.2 ± 0.35	306	**0.012**	29.9 ± 0.47	105	29.6 ± 0.33	285	0.477
	*C*	32.5 ± 0.37	300	32.6 ± 0.69	81	0.974	29.7 ± 0.33	310	29.7 ± 0.53	80	0.950
Pre-weaning growth rate (g/d)	*A*	300 ± 5	119	301 ± 4	262	0.814	265 ± 4	145	268 ± 3	245	0.462
	*B*	313 ± 6	75	298 ± 3	306	**0.023**	269 ± 4	105	266 ± 3	285	0.500
	*C*	300 ± 4	300	303 ± 7	81	0.762	268 ± 3	310	264 ± 5	80	0.445
Draft weight (kg)^2^	*A*	40.7 ± 0.38	119	40.7 ± 0.28	262	0.869					
	*B*	40.7 ± 0.47	75	40.7 ± 0.26	306	0.952					
	*C*	40.6 ± 0.27	300	40.8 ± 0.50	81	0.805					
Post-weaning growth rate (g/d)^2^	*A*	236 ± 13	119	227 ± 10	262	0.580					
	*B*	191 ± 16	75	239 ± 9	306	**0.005**					
	*C*	229 ± 9	300	234 ± 17	81	0.817					
Growth rate from birth to draft (g/d)^2^	*A*	283 ± 3	119	281 ± 2	262	0.548					
	*B*	280 ± 4	75	282 ± 2	306	0.499					
	*C*	276 ± 7	300	280 ± 8	82	0.456					

^1^Estimated means and standard errors from Linear Mixed-Models including “birth rank” as a fixed factor and “sire” as a random factor (*P* < 0.05 in bold).

^2^The draft weight, post-weaning growth rate and thus growth rate from birth to draft were only available for male lambs.

**Table 4:** Association of ovine *WFIKKN2*variants with carcass muscle traits (mean ± SE)^1^ in male NZ Romney lambs.

Carcass muscle trait	Variant being assessed	Other variants factored into in model	Variant absent	*n*	Variant present	*n*	*P* value^1^
H-W (kg)^2^	*A*	None	17.0 ± 0.23	119	16.9 ± 0.19	266	0.462
	*B*	None	16.6 ± 0.27	75	17.0 ± 0.18	310	0.189
	*C*	None	16.9 ± 0.18	303	16.8 ± 0.29	82	0.591
Leg yield (%)	*A*	None	21.6 ± 0.15	119	21.5 ± 0.12	266	0.639
	*B*	None	21.6 ± 0.18	75	21.6 ± 0.12	310	0.989
	*C*	None	21.6 ± 0.12	303	21.6 ± 0.19	82	0.878
Loin yield (%)	*A*	None	14.7 ± 0.11	119	14.5 ± 0.09	266	0.274
	*B*	None	14.5 ± 0.13	75	14.6 ± 0.09	310	0.525
	*C*	None	14.6 ± 0.09	303	14.6 ± 0.14	82	0.860
Shoulder yield (%)	*A*	None	17.0 ± 0.11	119	17.0 ± 0.09	266	0.899
	*B*	None	16.8 ± 0.13	75	17.0 ± 0.09	310	**0.043**
	*C*	None	17.0 ± 0.09	303	16.8 ± 0.14	82	0.288
Total yield (%)	*A*	None	53.2 ± 0.33	119	53.0 ± 0.27	266	0.522
	*B*	None	52.8 ± 0.38	75	53.2 ± 0.26	310	0.354
	*C*	None	53.1 ± 0.26	303	53.0 ± 0.42	82	0.722
Proportion leg yield (%)^†^	*A*	None	40.5 ± 0.11	119	40.6 ± 0.08	266	0.726
	*B*	None	40.8 ± 0.13	75	40.5 ± 0.07	310	0.057
	*C*	None	40.5 ± 0.08	303	40.7 ± 0.14	82	0.282
Proportion loin yield (%)^†^	*A*	None	27.6 ± 0.08	119	27.5 ± 0.06	266	0.263
	*B*	None	27.6 ± 0.11	75	27.5 ± 0.06	310	0.805
	*C*	None	27.5± 0.06	303	27.6 ± 0.11	82	0.399
Proportion shoulder yield (%)^†^	*A*	None	31.9 ± 0.11	119	31.9 ± 0.08	266	0.592
	*B*	None	31.7 ± 0.13	75	32.0 ± 0.07	310	0.035
	*C*	None	32.0 ± 0.08	303	31.7 ± 0.14	82	0.078
	*B*	*C*	31.6 ± 0.13	75	31.9 ± 0.09	310	0.095
	*C*	*B*	31.9 ± 0.09	303	31.7 ± 0.14	82	0.452

^1^Estimated means and standard errors from Linear Mixed-Models including “sire” as a random factor and “birth weight” as a covariate, except ^†^where “sire” was fitted as a random factor and “birth rank” as a fixed factor (*P* < 0.05 in bold).

^2^H-W = hot carcass weight. The weight of the carcass minus the pelt, head and gut.
